# Supplementary material for: Postmortem Electrical Conductivity Changes of Dicentrarchus labrax Skeletal Muscle: Root Mean Square (RMS) Parameter in Estimating Time since Death
Source: Animals (Basel). 2022 Apr 20;12(9):1062. doi: 10.3390/ani12091062 (PMC9105913; doi:10.3390/ani12091062)
Supplement: Supplementary file 1 [file animals-12-01062-s001.zip › animals-1676976-supplementary.pdf]

RMS	T1	T2	T3	T4	T5
SP 1	3,40	3,33	3,31	3,31	3,30
SP 2	2,45	2,45	2,44	2,43	2,43
SP 3	3,04	2,94	2,90	2,93	2,92
SP 4	3,62	3,54	3,53	3,53	3,55
SP 5	3,62	3,50	3,48	3,47	3,48
SP 6	2,95	2,86	2,84	2,81	2,82
SP 7	3,70	3,45	3,42	3,41	3,39
SP 8	4,04	3,94	3,93	3,92	3,93
SP 9	3,34	3,34	3,32	3,32	3,31
SP 10	2,93	2,93	2,84	2,94	2,93
SP 11	3,02	2,69	2,68	2,66	2,65
SP 12	3,58	3,43	3,42	3,41	3,39
SP 13	3,95	3,91	3,85	3,81	3,82
SP 14	3,38	3,30	3,24	3,23	3,23
SP 15	3,95	3,74	3,72	3,71	3,72
SP 16	2,61	2,61	2,58	2,57	2,57
SP 17	2,32	2,29	2,27	2,27	2,30
SP 18	3,48	3,42	3,38	3,34	3,32

T6	T7	T8	T9	T10	T11	T12
3,34	3,37	3,38	3,41	3,43	3,45	3,49
2,42	2,42	2,43	2,42	2,41	2,42	2,44
2,93	2,94	2,94	2,95	2,94	2,95	2,94
3,56	3,58	3,60	3,61	3,63	3,65	3,69
3,47	3,49	3,50	3,52	3,53	3,56	3,58
2,84	2,84	2,86	2,87	2,88	2,92	2,95
3,41	3,43	3,43	3,42	3,43	3,44	3,43
3,95	3,97	3,99	4,02	4,04	4,08	4,11
3,35	3,38	3,39	3,42	3,44	3,46	3,50
2,93	2,93	2,91	2,94	2,93	2,94	2,95
2,66	2,67	2,68	2,69	2,70	2,70	2,71
3,42	3,43	3,46	3,47	3,48	3,52	3,55
3,83	3,84	3,85	3,86	3,88	3,91	3,94
3,24	3,25	3,26	3,28	3,29	3,30	3,32
3,70	3,72	3,74	3,75	3,76	3,78	3,80
2,57	2,59	2,57	2,60	2,63	2,66	2,68
2,31	2,29	2,31	2,30	2,30	2,34	2,38
3,33	3,33	3,34	3,36	3,35	3,36	3,38

T13	T14	T15	T16	T17	T18	T19
3,52	3,55	3,58	3,59	3,59	3,60	3,59
2,44	2,44	2,46	2,49	2,49	2,49	2,50
2,96	2,98	2,99	3,01	3,02	3,03	3,04
3,72	3,72	3,73	3,75	3,74	3,73	3,73
3,60	3,64	3,64	3,66	3,66	3,70	3,71
2,97	2,98	2,99	3,00	2,99	3,02	3,01
3,44	3,44	3,46	3,48	3,49	3,49	3,51
4,13	4,12	4,14	4,15	4,14	4,15	4,14
3,51	3,56	3,59	3,60	3,59	3,61	3,59
3,01	3,01	3,01	3,02	3,01	3,04	3,03
2,72	2,74	2,77	2,80	2,81	2,83	2,85
3,58	3,61	3,63	3,66	3,66	3,67	3,65
3,96	3,97	3,98	4,00	4,00	4,01	4,02
3,32	3,34	3,34	3,33	3,35	3,34	3,35
3,83	3,85	3,86	3,88	3,87	3,90	3,88
2,70	2,75	2,73	2,77	2,79	2,80	2,80
2,37	2,39	2,40	2,39	2,40	2,43	2,42
3,39	3,40	3,40	3,41	3,40	3,41	3,40

T20	T21	T22	T23	T24	T25	T26
3,60	3,63	3,61	3,64	3,65	3,63	3,63
2,51	2,51	2,54	2,56	2,56	2,56	2,58
3,03	3,05	3,04	3,05	3,04	3,04	3,04
3,74	3,75	3,74	3,75	3,76	3,75	3,75
3,73	3,75	3,76	3,78	3,79	3,79	3,78
3,03	3,02	3,02	3,03	3,04	3,04	3,04
3,51	3,53	3,54	3,56	3,56	3,57	3,58
4,11	4,16	4,16	4,16	4,15	4,15	4,16
3,61	3,68	3,63	3,62	3,64	3,62	3,64
3,06	3,04	3,04	3,05	3,05	3,05	3,06
2,86	2,88	2,90	2,91	2,92	2,92	2,93
3,64	3,64	3,66	3,65	3,66	3,67	3,67
4,03	4,02	4,03	4,03	4,04	4,04	4,05
3,36	3,36	3,36	3,37	3,36	3,37	3,38
3,90	3,96	3,97	3,99	4,01	4,04	4,05
2,81	2,84	2,86	2,87	2,88	2,90	2,89
2,44	2,44	2,46	2,42	2,41	2,43	2,47
3,41	3,42	3,42	3,44	3,43	3,44	3,46

T27	T28	T29	T30	T31	T32	T33
3,64	3,63	3,60	3,61	3,58	3,60	3,60
2,60	2,61	2,64	2,64	2,65	2,64	2,66
3,05	3,06	3,05	3,06	3,05	3,06	3,07
3,76	3,73	3,74	3,73	3,73	3,71	3,71
3,81	3,82	3,84	3,85	3,85	3,87	3,87
3,05	3,04	3,05	3,05	3,06	3,07	3,06
3,60	3,62	3,64	3,64	3,65	3,66	3,67
4,16	4,16	4,16	4,18	4,16	4,17	4,16
3,63	3,64	3,61	3,61	3,59	3,61	3,60
3,06	3,05	3,06	3,06	3,06	3,06	3,06
2,94	2,96	2,95	2,96	2,95	2,96	2,95
3,68	3,68	3,68	3,68	3,70	3,70	3,70
4,06	4,05	4,05	4,06	4,06	4,06	4,07
3,37	3,38	3,38	3,39	3,41	3,40	3,41
4,08	4,11	4,12	4,13	4,13	4,13	4,12
2,92	2,96	2,98	2,99	2,98	3,01	3,00
2,48	2,49	2,47	2,43	2,45	2,48	2,44
3,47	3,48	3,49	3,50	3,49	3,51	3,50

T34	T35	T36	T37	T38	T39	T40
3,58	3,58	3,59	3,58	3,57	3,57	3,56
2,65	2,66	2,66	2,67	2,66	2,64	2,65
3,08	3,07	3,08	3,08	3,09	3,09	3,08
3,70	3,70	3,68	3,67	3,67	3,66	3,66
3,86	3,88	3,88	3,89	3,88	3,88	3,88
3,06	3,07	3,07	3,08	3,09	3,09	3,09
3,69	3,68	3,66	3,65	3,66	3,65	3,65
4,17	4,17	4,19	4,18	4,18	4,18	4,18
3,60	3,60	3,60	3,58	3,59	3,57	3,59
3,07	3,07	3,08	3,07	3,08	3,08	3,08
2,94	2,95	2,95	2,94	2,94	2,94	2,93
3,70	3,69	3,68	3,68	3,67	3,67	3,67
4,06	4,07	4,07	4,08	4,08	4,09	4,09
3,38	3,37	3,36	3,37	3,35	3,34	3,34
4,12	4,11	4,12	4,11	4,11	4,10	4,11
3,00	3,01	3,03	3,03	3,04	3,03	3,03
2,45	2,41	2,43	2,43	2,48	2,46	2,43
3,51	3,50	3,49	3,47	3,48	3,47	3,46

T41	T42	T43	T44	T45	T46	T47
3,55	3,55	3,56	3,54	3,53	3,53	3,54
2,65	2,65	2,65	2,65	2,63	2,63	2,62
3,08	3,09	3,09	3,08	3,09	3,09	3,09
3,65	3,65	3,66	3,65	3,63	3,63	3,64
3,87	3,87	3,85	3,86	3,85	3,85	3,85
3,10	3,09	3,10	3,09	3,10	3,10	3,11
3,65	3,64	3,65	3,64	3,64	3,63	3,63
4,19	4,21	4,21	4,20	4,21	4,21	4,22
3,56	3,56	3,57	3,55	3,54	3,54	3,53
3,09	3,07	3,09	3,08	3,07	3,09	3,09
2,91	2,93	2,92	2,93	2,92	2,91	2,90
3,67	3,66	3,65	3,63	3,64	3,63	3,62
4,10	4,09	4,10	4,10	4,11	4,11	4,11
3,33	3,32	3,32	3,29	3,30	3,31	3,32
4,10	4,10	4,09	4,09	4,09	4,08	4,09
3,04	3,04	3,05	3,04	3,04	3,05	3,06
2,44	2,41	2,45	2,47	2,43	2,43	2,42
3,47	3,46	3,45	3,46	3,45	3,45	3,44

T48	T49	T50	T51	T52	T53	T54
3,53	3,53	3,52	3,52	3,51	3,51	3,52
2,61	2,60	2,61	2,60	2,60	2,59	2,58
3,10	3,09	3,11	3,10	3,11	3,10	3,10
3,63	3,63	3,62	3,61	3,62	3,61	3,62
3,85	3,84	3,85	3,85	3,85	3,84	3,85
3,11	3,11	3,11	3,11	3,11	3,11	3,10
3,61	3,60	3,61	3,59	3,59	3,57	3,58
4,23	4,23	4,23	4,24	4,25	4,25	4,24
3,54	3,52	3,54	3,53	3,53	3,52	3,53
3,09	3,10	3,10	3,10	3,10	3,11	3,09
2,90	2,89	2,89	2,90	2,90	2,89	2,88
3,62	3,62	3,60	3,61	3,60	3,60	3,58
4,13	4,13	4,14	4,14	4,13	4,13	4,12
3,32	3,33	3,32	3,34	3,27	3,26	3,27
4,08	4,06	4,07	4,07	4,06	4,07	4,07
3,05	3,06	3,06	3,06	3,07	3,06	3,06
2,44	2,39	2,39	2,42	2,41	2,40	2,40
3,44	3,44	3,42	3,42	3,41	3,42	3,42

T55	T56	T57	T58	T59	T60	T61
3,50	3,50	3,51	3,50	3,48	3,48	3,47
2,60	2,58	2,58	2,58	2,57	2,56	2,55
3,10	3,11	3,09	3,10	3,09	3,09	3,10
3,61	3,60	3,61	3,60	3,59	3,60	3,59
3,83	3,83	3,83	3,84	3,83	3,80	3,81
3,10	3,10	3,10	3,10	3,10	3,10	3,11
3,57	3,58	3,58	3,57	3,56	3,57	3,55
4,23	4,24	4,24	4,24	4,23	4,23	4,22
3,51	3,51	3,51	3,52	3,51	3,48	3,49
3,10	3,10	3,10	3,10	3,10	3,09	3,11
2,89	2,87	2,87	2,87	2,86	2,86	2,85
3,58	3,57	3,57	3,58	3,56	3,56	3,56
4,12	4,12	4,12	4,11	4,10	4,10	4,11
3,26	3,25	3,26	3,25	3,25	3,25	3,24
4,07	4,05	4,05	4,05	4,04	4,04	4,05
3,06	3,06	3,06	3,06	3,04	3,04	3,03
2,38	2,44	2,41	2,38	2,42	2,38	2,42
3,41	3,41	3,40	3,39	3,39	3,40	3,39

T62	T63	T64	T65	T66	T67	T68
3,47	3,48	3,47	3,48	3,47	3,46	3,47
2,56	2,55	2,55	2,55	2,55	2,55	2,54
3,09	3,08	3,09	3,08	3,09	3,09	3,09
3,57	3,57	3,58	3,57	3,57	3,57	3,56
3,81	3,80	3,80	3,80	3,80	3,80	3,78
3,10	3,10	3,10	3,10	3,10	3,08	3,08
3,56	3,55	3,55	3,55	3,56	3,55	3,53
4,21	4,21	4,21	4,21	4,22	4,21	4,22
3,50	3,48	3,48	3,48	3,48	3,48	3,46
3,09	3,10	3,08	3,08	3,08	3,09	3,10
2,85	2,86	2,84	2,84	2,84	2,83	2,83
3,55	3,56	3,55	3,56	3,55	3,55	3,54
4,10	4,10	4,10	4,10	4,09	4,09	4,08
3,23	3,24	3,23	3,23	3,24	3,23	3,23
4,04	4,03	4,03	4,03	4,02	4,02	4,00
3,03	3,03	3,01	3,01	3,04	3,03	3,03
2,41	2,39	2,34	2,40	2,40	2,37	2,37
3,38	3,38	3,37	3,38	3,37	3,37	3,36

T69	T70	T71	T72	T73	T74	T75
3,45	3,46	3,45	3,44	3,44	3,43	3,44
2,53	2,54	2,54	2,53	2,52	2,52	2,53
3,10	3,08	3,09	3,08	3,08	3,07	3,07
3,56	3,57	3,56	3,56	3,55	3,56	3,55
3,76	3,76	3,75	3,75	3,75	3,75	3,73
3,09	3,08	3,08	3,09	3,07	3,08	3,07
3,54	3,53	3,53	3,52	3,52	3,51	3,52
4,23	4,22	4,22	4,21	4,21	4,21	4,20
3,45	3,45	3,44	3,45	3,44	3,45	3,43
3,10	3,09	3,09	3,10	3,08	3,08	3,07
2,82	2,82	2,82	2,82	2,83	2,82	2,82
3,54	3,54	3,53	3,54	3,53	3,53	3,53
4,09	4,08	4,08	4,09	4,08	4,07	4,07
3,23	3,22	3,22	3,23	3,21	3,22	3,21
4,00	3,99	3,98	3,98	3,98	3,97	3,98
3,02	3,02	3,00	3,01	2,99	2,99	2,98
2,39	2,35	2,33	2,37	2,30	2,36	2,34
3,37	3,36	3,36	3,36	3,34	3,35	3,34

T76	T77	T78	T79	T80	T81	T82
3,43	3,43	3,42	3,42	3,42	3,41	3,40
2,51	2,52	2,51	2,51	2,51	2,51	2,50
3,08	3,06	3,08	3,07	3,07	3,08	3,07
3,54	3,54	3,53	3,52	3,53	3,52	3,52
3,74	3,72	3,70	3,70	3,70	3,69	3,69
3,08	3,08	3,05	3,06	3,06	3,06	3,05
3,51	3,51	3,51	3,51	3,50	3,49	3,49
4,21	4,20	4,20	4,17	4,20	4,18	4,17
3,45	3,43	3,45	3,43	3,43	3,42	3,43
3,08	3,08	3,08	3,06	3,08	3,08	3,07
2,82	2,81	2,82	2,81	2,81	2,81	2,80
3,52	3,53	3,53	3,52	3,52	3,52	3,52
4,07	4,06	4,05	4,06	4,05	4,05	4,05
3,22	3,22	3,21	3,21	3,21	3,20	3,21
3,97	3,97	3,97	3,96	3,96	3,96	3,95
2,98	2,99	2,99	2,99	2,99	2,98	2,98
2,33	2,27	2,30	2,34	2,32	2,31	2,31
3,34	3,33	3,34	3,33	3,33	3,32	3,32

T83	T84	T85	T86	T87	T88	T89
3,41	3,41	3,41	3,40	3,40	3,39	3,40
2,50	2,49	2,48	2,48	2,48	2,48	2,48
3,06	3,06	3,06	3,05	3,06	3,05	3,05
3,51	3,51	3,50	3,51	3,50	3,49	3,48
3,68	3,68	3,68	3,67	3,67	3,68	3,67
3,05	3,05	3,04	3,04	3,03	3,03	3,04
3,48	3,48	3,48	3,48	3,47	3,48	3,47
4,17	4,16	4,15	4,15	4,15	4,14	4,15
3,43	3,43	3,42	3,42	3,42	3,41	3,40
3,07	3,07	3,06	3,06	3,05	3,05	3,06
2,81	2,80	2,80	2,79	2,79	2,78	2,79
3,50	3,52	3,50	3,51	3,50	3,50	3,49
4,04	4,04	4,03	4,04	4,03	4,03	4,03
3,20	3,20	3,20	3,20	3,19	3,20	3,19
3,95	3,95	3,93	3,93	3,91	3,92	3,91
2,98	2,99	2,98	2,98	2,98	2,98	2,98
2,28	2,31	2,25	2,27	2,30	2,26	2,30
3,31	3,30	3,31	3,30	3,31	3,30	3,29

T90	T91	T92	T93	T94	T95	T96
3,39	3,39	3,40	3,39	3,40	3,39	3,39
2,47	2,49	2,48	2,47	2,47	2,46	2,47
3,04	3,04	3,03	3,02	3,02	3,03	3,02
3,48	3,49	3,48	3,48	3,47	3,38	3,48
3,68	3,67	3,67	3,67	3,66	3,67	3,67
3,03	3,02	3,02	3,00	3,01	3,00	3,00
3,47	3,47	3,46	3,46	3,45	3,45	3,45
4,13	4,13	4,13	4,12	4,12	4,13	4,12
3,41	3,42	3,42	3,40	3,41	3,41	3,42
3,05	3,04	3,04	3,03	3,02	3,04	3,01
2,78	2,78	2,77	2,78	2,77	2,77	2,77
3,49	3,48	3,50	3,49	3,48	3,48	3,47
4,02	4,01	4,01	4,00	4,01	4,00	4,00
3,19	3,19	3,18	3,19	3,19	3,19	3,18
3,90	3,88	3,87	3,87	3,86	3,87	3,86
2,98	2,98	2,98	2,96	2,96	2,98	2,97
2,23	2,27	2,27	2,26	2,26	2,26	2,19
3,29	3,28	3,29	3,28	3,28	3,27	3,28
